# Activation Mechanism of Protein Kinase B by DNA-dependent Protein Kinase Involved in the DNA Repair System

**DOI:** 10.5487/TR.2008.24.3.175

**Published:** 2008-09-01

**Authors:** Yuwen Li, Longzhen Piao, Keum-Jin Yang, Sanghee Shin, Eulsoon Shin, Kyung Ah Park, Hee Sun Byun, Minho Won, Byung Lyul Choi, Hyunji Lee, Young-Rae Kim, Jang Hee Hong, Gang Min Hur, Jeong-Lan Kim, Jae Youl Cho, Jeong Ho Seok, Jongsun Park

**Affiliations:** 13 Department of Pharmacology, Cell Signaling Laboratory, Research Center for Transgenic Cloned Pigs, Daejeon Regional Cancer Center, Cancer Research Institute, Research Institute for Medical Sciences, Korea; 23 Department of Psychiatry, College of Medicine, Chungnam National University, Taejeon, 301-131 Korea; 33 School of Bioscience and Biotechnology, and Institute of Bioscience and Biotechnology, Kangwon National University, Chuncheon, 200-701 Korea; 43 Department of Pharmacology, College of Medicine, Chungnam National University, Taejeon, 301-131 Korea

**Keywords:** DNA-PK, DNA damage, Protein kinase B, Insulin signaling, Cell Signaling

## Abstract

DNA-dependent protein kinase (DNA-PK) is involved in joining DNA double-strand breaks induced by ionizing radiation or V(D)J recombination and is activated by DNA ends and composed of a DNA binding subunit, Ku, and a catalytic subunit, DNA-PKcs. It has been suggested that DNA-PK might be 2^nd^ upstream kinase for protein kinase B (PKB). In this report, we showed that Ser473 phosphorylation in the hydrophobic-motif of PKB is blocked in DNA-PK knockout mouse embryonic fibroblast cells (MEFs) following insulin stimulation, while there is no effect on Ser473 phosphorylation in DNA-PK wild type MEF cells. The observation is further confirmed in human glioblastoma cells expressing a mutant form of DNA-PK (M059J) and a wild-type of DNA-PK (M059K), indicating that DNA-PK is indeed important for PKB activation. Furthermore, the treatment of cells with doxorubicin, DNA-damage inducing agent, leads to PKB phosphorylation on Ser473 in control MEF cells while there is no response in DNA-PK knockout MEF cells. Together, these results proposed that DNA-PK has a potential role in insulin signaling as well as DNA-repair signaling pathway.

## INTRODUCTION

Over time, DNA accumulates changes that activate proto-oncogenes and inactivate tumor-suppressor genes. The genetic instability driving tumorigenesis is fuelled by DNA damage and by errors made by the DNA machinery. However, 'spontaneous' mutations are insufficient to explain the lifetime cancer risk (Loeb, 1991). Indeed, numerous links have been identified between oncogenesis and acquired or inherited faulty genome guardians that cause a 'mutator' phenotype, highlighting the key role of DNA protection systems in tumor prevention (Hoeijmakers, 2001b). To prevent or counteract the time-dependent erosion of our genes due to undesired but irresistible accumulation of DNA injury one of the most important systems is DNA repair. Because there are so many different types of damage, it is simply impossible that one mechanism is able to cope with all of them. So, every repair system has its own tasks, its own spectrum of damage that it can recognize and fix and other repair systems will deal with other types of damage. But, as a whole, a network of repair systems will be able to cope with almost every possible lesion in the DNA (Citterio *et al.*, 2000).

DNA double-strand breaks (DSBs) can be accidentally introduced in cells by the action of ionizing radiation or certain reactive radicals. These agents have the ability to initiate a series of chemical reactions that ultimately sever the DNA backbone, resulting in chromosome breakage and fragmentation of genes (Hoeijmakers, 2001). Because such corruption of genetic material inevitably leads to problems with replication and cell division, it is of the utmost importance that cells have a mechanism to counteract DSBs (van Gent *et*

[#]These authors contributed equally to this work.

*al.*, 2001). In addition, DSBs are generated in developing B and T cells during normal V(D)J recombination, implying that a working DSB repair system is not only necessary for an effective defense against DNA-modifying agents but also for a functional immune system in higher organisms (Weterings and van Gent, 2004). As a result, two highly efficient DSB repair pathways have evolved in eukaryotic cells: homologous recombination (HR) and nonhomologous end joining (NHEJ) (Weterings and Chen, 2007). The core protein of the DNA-PK multi-protein complex is represented by a Mr ~460,000 catalytic subunit (DNA-PKcs) which, in itself, is a serine-threonine kinase similar to proteins of the PI3K family (Hartley *et al.*, 1995; Meek *et al.*, 2004).

The serine/threonine kinase PKB (protein kinase B, also called Akt) constitutes an important node in diverse signaling cascades and acts as a major signal transducer downstream of activated PI3K (phosphoinositide 3-kinase) (Dummler and Hemmings, 2007). Mammalian cells contain three genes that encode three closely related and highly conserved isoforms of PKB, termed PKBα/Akt1, PKBβ/Akt2, and PKBγ/Akt3 (Yang *et al.*, 2004). Stimulation by numerous growth factors, cytokines, hormones and neurotransmitters can activate PKB/Akt in a phosphoinositide 3-kinase-dependent manner (Brazil *et al.*, 2004). Through receptor tyrosine kinases, these stimuli cause phosphoinositide 3-kinase activation, and generation of the membrane phospholipid PtdIns(3,4,5)P3. PtdIns(3,4,5)P3 then recruits PKB/Akt to the membrane, where it becomes phosphorylated at Thr308 and Ser473 (for PKBα/Akt1) by two upstream kinases, phosphoinositide-dependent kinase 1 and a yet to be identified Ser473 kinase. The unphosphorylated form of PKB is virtually inactive, and PDK1 phosphorylation stimulates its activity by at least 100-fold (Alessi *et al.*, 1996, 1997; Stephens *et al.*, 1998; Stokoe *et al.*, 1997). Furthermore, there is a second regulatory site, Ser473 at the carboxyl terminus, termed the hydrophobic motif which is responsible for further 7- to 10-fold full-activation of PKB (Alessi *et al.*, 1996). Recently, it has been shown that DNA-dependent protein kinase (DNA-PK) is a long sought after kinase, responsible for the Ser473 phosphorylation step in the activation of PKB (Feng *et al.*, 2004).

In the current report, we further provided the evidence that DNA-PK is a Ser473 kinase responsible for PKB activation by using DNA-PK knockout MEF cells.

## MATERIALS AND METHODS

**Reagents and antibodies.** Anti-PKB antibody (Ab10) has been described previously (Andjelkovic *et al.*, 1997). Anti-phospho specific Thr308 or Ser473 (PKB) antibodies are from Cell Signaling. Anti-DNA-PKcs (G4) antibody is obtained from Santa Cruz Biotech. Double-stranded fetal calf thymus DNA was from Sigma. The purified DNA-PKcs, Ku70/80 and DNA-PK substrate peptide were purchased from Promega. Inositol hexakisphosphate, Spermine, poly-L-lysine, Heparine, and poly (Glu-Na, Tyr) 4:1 were purchased from Sigma.

**Cell culture and stimulation.** M059J and M059K were from ATCC. DNA-PK wild type mouse embryonic fibroblast cells (MEFs), DNA-PK knockout MEFs, M059K cells, M059J cells, ataxia telangiectasia mutated (ATM) wild type MEFs and ATM knockout MEFs were maintained in DMEM supplemented with 10% FCS, 1 mM non-essential amino acid (Gibco) and 20 mM L-Glutamine (Gibco). And cells were serum-starved for 24 h before stimulation with 100 nM insulin (Boehringer Mannheim) or 5 μM doxorubicin (Sigma) for the indicated times.

***In vitro* kinase assays.** *In vitro* DNA-PK assays were performed for 15 min at 30°C. Briefly, 1.25 μg/ml of DNA-PKcs was assayed with or without 100 μg/ml of DNA and 1.75 μg/ml of Ku subunits, 1.0 mg/ml substrate peptide, 1 μM protein kinase A inhibitor peptide (PKI; Bachem), and 50 μM [$\gamma$-$^{32}$P]ATP (Amersham; 1,000~2,000 cpm/pmol) in Z'0.05 buffer (25 mM HEPES pH 7.5, 50 mM KCl, 10 mM $MgCl_2$, 20% glycerol, 1% w/v Nonidet P-40 and 1 mM dithiothreitol) (Beamish *et al.*, 2000) as indicated in each experiment. All reactions were stopped by adding 50 μM EDTA and processed as described previously (Park *et al.*, 2001). Protein concentrations were determined by the method of Bradford (Bradford, 1976) using BSA as a standard.

**Immunoblot analysis.** Cell extracts were resolved by 6% or 12% SDS/PAGE and transferred to Immobilon P membranes (Millipore). The membranes were blocked for 30 min in 1 × PBS containing 5% skimmed milk, and 0.1% Triton X-100, followed by 2 h incubation with the first antibody diluted 1000-fold in the same blocking solution. The secondary antibody was a goat anti-rabbit or anti-mouse IgG HRP-conjugated antibody (Amersham Biosciences) diluted 2500-fold in the blocking buffer. The detection and quantitation of protein expression were developed by using the ECL development reagents (Pharmacia) and exposure to film.

**Data analysis.** The results from western blot analysis are a representative of three independent experiments. Kinase activity is the average (± SD) of three independent experiments.

## RESULTS

**The absence of Ser473 PKB kinase activity from DNA-PK knockout MEF cells.** It has been suggested that DNA-PK act as an upstream kinase for PKB through Ser473 phosphorylation (Feng *et al.*, 2004). Numerous studies have proposed that DNA-PK is involved in DNA-repair as well as V(D)J recombination (Hoeijmakers, 2001a). During last decade, several substrates for DNA-PK have been suggested in vitro as well as *in vivo* (Burma and Chen, 2004; Meek *et al.*, 2004). However, the significance of those substrates is not well established. During the purification of Ser473 kinase from the membrane fraction of HEK 293 cells, in which the mechanism of PKB activation is well established (Andjelkovic *et al.*, 1997, 1999), it has been found that DNA-PK as a Ser473 kinase (Feng *et al.*, 2004). Therefore, we monitored the status of Ser473 phosphorylation of PKB in DNA-PK knockout MEFs (Taccioli *et al.*, 1998) following the treatment of cells with insulin. As shown in Fig. 1, DNA-PK knock-out MEFs didn't properly respond to insulin stimulation in Ser473 phosphorylation of PKB whereas DNA-PK wild type control MEFs show a nice increase in Ser473 phosphorylation in a time-dependent manner. Thus these results indicate that DNA-PK is essential for PKB phosphorylation on Ser 473 and activation.

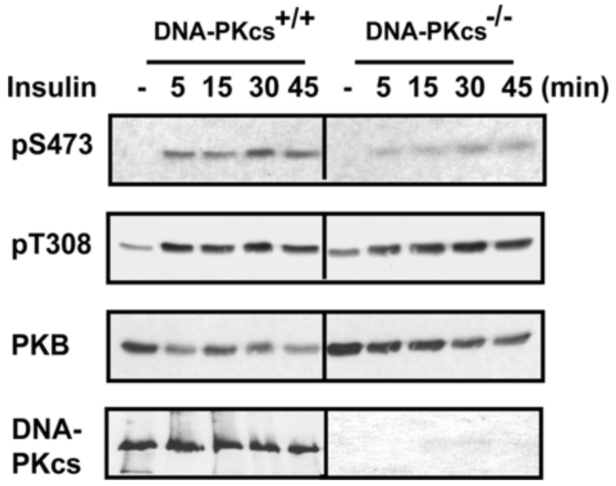

**Fig. 1.** The status of PKB activation in DNA-PK knockout mouse embryonic fibroblast (MEF) cells. DNA-PKcs wild type (+/+) and knockout (-/-) MEFs were treated with 100 nM insulin in the indicated time after 18 hour serum-starvation. Then 50 μg of cell lysate were subjected to 6% or 12% SDS-PAGE and immunoblotted with pS473 or pT308 phospho-specific antibodies, anti-PKB antibodies or anti-DNA-PK antibodies. Each result is a representative of three independent experiments.

**Lack of detection in Ser473 phosphorylation of PKB in human glioblastoma cells expressing an inactive mutant form of DNA-PK, M059J cells.** In order to further develop the previous finding that DNA-PK is responsible for Ser473 phosphorylation, we take the advantage of human glioblastoma cells expressing an inactive mutant form of DNA-PK (M059J) or an active wild type of DNA-PK (M059K) (Allalunis-Turner *et al.*, 1993). As expected, Ser473 phosphorylation of PKB was impaired in M059J cells, but not in wild type control cells, M059K cells (Fig. 2), confirming that Ser473 phosphorylation of PKB is mediated by DNA-PK.

**PKB activation is normal in ATM knockout MEF cells as well as ATM wild type MEF cells.** Recently, Viniegra and colleagues provide new clues in the search of the unknown Ser473 kinase that full activation of PKB/Akt in response to insulin or ionizing radiation is mediated through ATM (Viniegra *et al.*, 2005). Therefore we also monitored the status of Ser473 phosphorylation of PKB in ATM knockout MEFs together with ATM wild type MEFs. Contrast with the previous results, PKB activation in response to insulin treatment was increased in ATM knockout MEFs as well as ATM wild type control MEFs (Fig. 3).

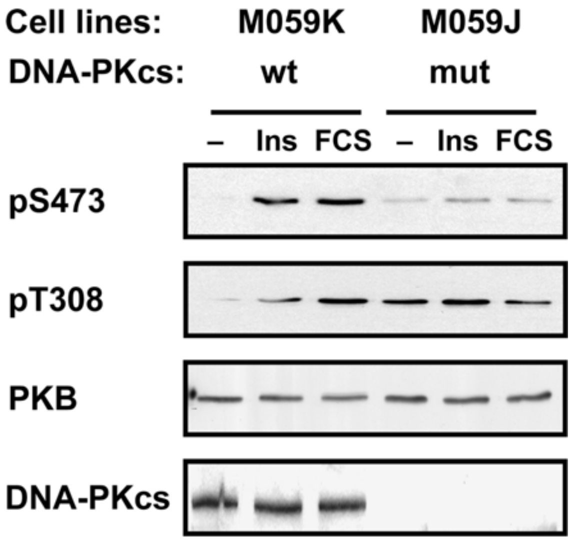

**Fig. 2.** Inhibition of insulin-mediated PKB activation in DNA-PK-deficient human glioblastoma cell lines. Human glioblastoma cells expressing a wild type DNA-PK, M059K or an inactive form of DNA-PK, M059J cells were treated with 100 nM insulin in the indicated time after 18 hour serum-starvation. Then 50 μg of cell lysate were subjected to 6% or 12% SDS-PAGE and immunoblotted with pS473 or pT308 phospho-specific antibodies, anti-PKB antibodies or anti-DNA-PK antibodies. Each result is a representative of three independent experiments.

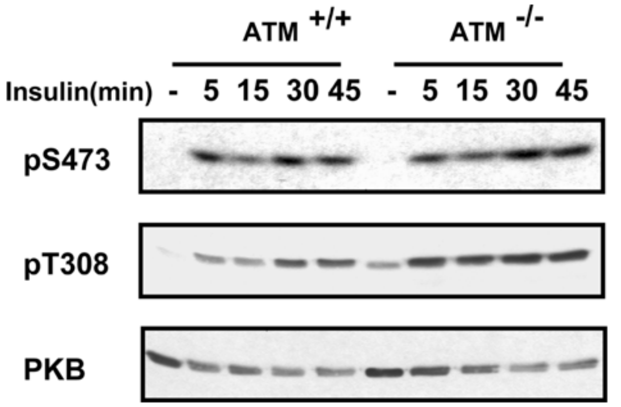

**Fig. 3.** Insulin-induced PKB activation in ATM knockout MEF cells. ATM wild type (+/+) and knockout (-/-) MEFs were treated with 100 nM insulin in the indicated time and then analyzed by immunoblot analysis. Each result is a representative of three independent experiments.

**Doxorubicin, a DNA-damage inducing agent, mediated Ser473 phosphorylation of PKB is inhibited in DNA-PK knockout MEF cells.** Since DNA-PK play a major role in DNA-repairing system (Meek *et al.*, 2004), we further examined the effects of doxorubicin, DNA-damage inducing agent, on Ser473 phosphorylation of PKB. Consistent with previous reports (Li *et al.*, 2005; Quevedo *et al.*, 2007), Ser473 phosphorylation of PKB is diminished in DNA-PK knockout MEFs whereas there is a gradual increase in Ser473 phosphorylation in a time-dependent manner in DNA-PK wild type control MEFs (Fig. 4A). This effect was not observed in ATM knockout MEFs (Fig. 4B), indicating that PKB activation by DNA-PK could also occur in DNA-damage condition.

**Inositol hexakisphosphate do not activate DNA-PK in presence of Ku70/Ku86.** To further evaluate the effect of DNA-PK on PKB activation, we have screened the potential activator of DNA-PK. Inositol hexakisphosphate (IP6) has been found as a novel factor that is bound by DNA-PK and stimulates DNA end-joining in vitro (Byrum *et al.*, 2004; Hanakahi *et al.*, 2000; Hanakahi and West, 2002; Ma and Lieber, 2002). Therefore, we use IP6 for modulating DNA-PK activity in vitro in presence/absence of Ku70/Ku86 subunit. As shown in Fig. 5A, double-strand (ds) DNA is nicely able to induce the DNA-PK activity toward FSYtide whereas different concentration of IP6 failed to activate DNA-PK. Additionally, we also tested some of charged molecule which mimic the negative charge of dsDNA to activate DNA-PK activity such as Spermine, Poly-L-lysine, Poly-Glu-Tyr, and Heparin. However, none of them success to activate DNA-PK activity toward FSYtide in presence of Ku70/Ku86 (Fig. 5B). Taken together, we were not able to find the modulator of DNA-PK activity addition to dsDNA.

**Differential regulation of DNA-PK activity by $Mg^{2+}$ and $Mn^{2+}$.** It has been reported that DNA-PK activity toward degenerative non-SQ peptide library can be increased in presence of $Mn^{2+}$ instead of $Mg^{2+}$ in in-vitro kinase assay (O'Neill *et al.*, 2000). In addition, Chan et al found that DNA-PK activity is independent on double-strand (ds) DNA ends and Ku70/80 subunit in presence of $Mn^{2+}$ whereas DNA-PK activity is dependent on dsDNA ends and Ku70/80 subunit in presence of $Mg^{2+}$ (Chan *et al.*, 2000), suggesting the possibility of differential regulation of DNA-PK activity by $Mg^{2+}$ and $Mn^{2+}$. Therefore we test the DNA-PK activity toward FSYtide or p53tide with either $Mg^{2+}$ or $Mn^{2+}$ in *in-vitro* kinase reaction. Apparently, DNA-PK activity was not changed so much in these conditions (data not shown).

## DISCUSSION

In higher eukaryotes, non-homologous end-joining

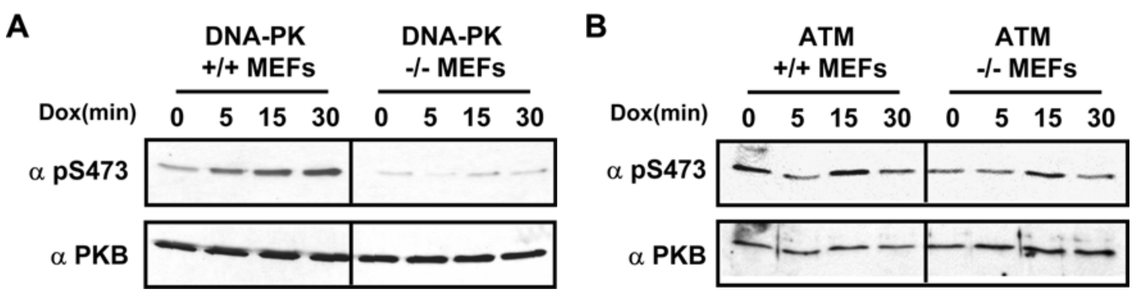

**Fig. 4.** The effects of Doxorubicin, DNA-damage inducing agent, on PKB activity in DNA-PK knockout MEF cells and ATM knockout MEF cells. (A) DNA-PK wild type (+/+), knockout (-/-) MEFs, (B) ATM wild type (+/+) and knockout (-/-) MEFs were treated with 30 μM Doxorubicin in the indicated time after 18 hour serum-starvation. Then 50 μg of cell lysate were subjected to 6% or 12% SDS-PAGE and immunoblotted with pS473 phospho-specific antibodies or anti-PKB antibodies. Each result is a representative of three independent experiments.

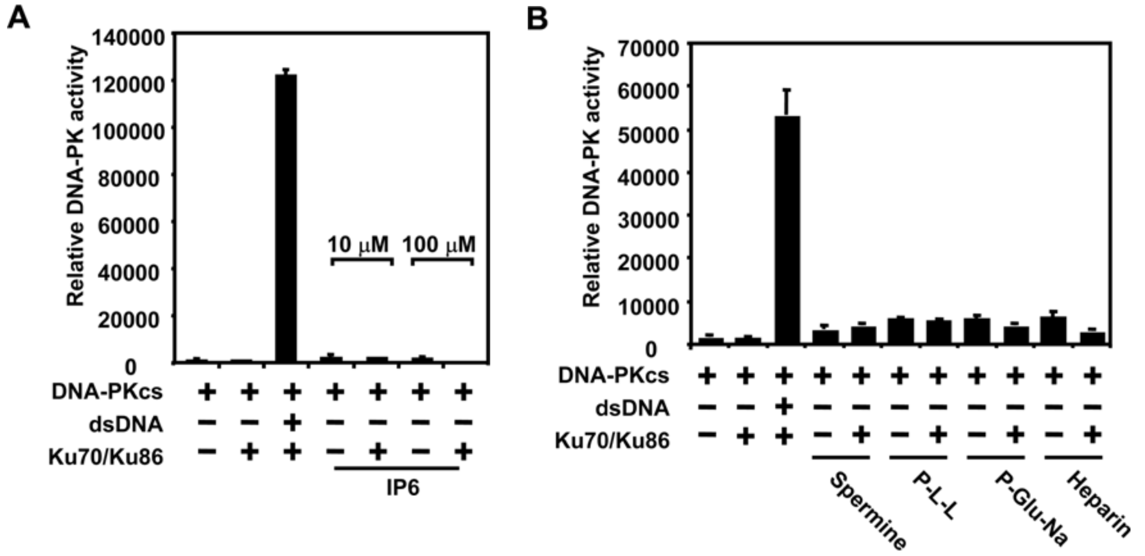

**Fig. 5.** The effects of InsP6 and polyions on DNA-PK activity toward FSYtide *in vitro*. (A) DNA-PK activity was assayed using FSYtide peptide as substrate with (+) or without (-) Ku subunit in the presence of different concentration of IP6 as indicated. (B) DNA-PK activity was assayed with (+) or without (-) Ku subunit in the presence of 0.2 mg/ml Spermine (Sper), poly-L-lysine (PLL), poly (Glu-Na, Tyr) 4:1 (P-Glu-Na) and Heparine (Hep). Activity of DNA-PK Kinase activity is the average (± SD) of three independent experiments.

(NHEJ) DNA is the primary pathway that repairs these breaks. NHEJ also functions in developing lymphocytes to repair strand breaks that occur during V(D)J recombination, the site-specific recombination process that provides for the assembly of functional antigen-receptor genes (Meek *et al*., 2004). In the last decade, an intensive research effort has focused on NHEJ resulting in a reasonable understanding of how double-strand breaks are resolved. Six distinct gene products have been identified that function in this pathway (Ku70, Ku86, XRCC4, DNA ligase IV, Artemis, and DNA-PKcs). Three of these comprise one complex, the DNA-dependent protein kinase (DNA-PK). This protein complex is central during NHEJ, because DNA-PK initially recognizes and binds to the damaged DNA and then targets the other repair activities to the site of DNA damage (Weterings and Chen, 2007). A large body of evidence suggested that activation of PI3K/PKB pathway is associated with resistance to radiation in many cell lines (Gottschalk *et al*., 2005; Grana *et al*., 2002; Gupta *et al*., 2001, 2003; Li *et al*., 2004). Therefore, it is tempting to speculate that radiation-induced DNA damage will result in activating PI3K/PKB signaling pathway through DNA-PK, ATM and ATR.

In current report, we have clearly demonstrated that DNA-PK is a key regulator to control PKB activation in insulin and DNA-repair signaling. Immunoblot analysis of DNA-PK knockout MEF cells suggested that PKB phosphorylation on Ser473 is significantly inhibited in DNA-PK knockout MEF cells following the treatment of cells with insulin (Fig. 1). These finding was further supported by the observation that PKB activation is blocked in human glioblastoma cells expressing an inactive mutant form of DNA-PK, M059J cells while PKB activation is normal in control M059K cells (Fig. 2). In addition, the treatment of cells with doxorubicin, a DNA-damage inducing agent, increased Ser473 phosphorylation of PKB in DNA-PK wild type MEFs, but not in DNA-PK knockout MEFs (Fig. 4A), suggesting that DNA-PK play a new role in PKB signaling.

Our results from ATM (-/-) MEFs (Fig. 3) contrast with the observation of Viniegra *et al*., who claimed that ATM is a major determinant of full PKB/Akt activation in response to insulin or gamma-radiation (Viniegra *et al*., 2005). However, ATM knockout MEFs in this report was generated from mice of p53 knockout background. Therefore, this discrepancy of observation is due to the fact that p53 somehow play a role in Ser473 phosphorylation of PKB in ATM knockout MEFs through DNA-PK in insulin signaling as well as DNA-repair signaling (Fig. 3 and 4B). Additionally, it has been proposed that mammalian target of rapamycin (mTOR) and its associated protein rictor are involved in Ser473 phosphorylation of PKB in Drosophila and human cells (Hresko and Mueckler, 2005; Sarbassov *et al*., 2005). Recently, Caporali and colleagues also provided the evidence of a novel function of ATR as an upstream activator of PKB in response to DNA damage induced by $O^6$-guanine methylating agents (Caporali *et al.*, 2008).

Toker and colleagues suggested that phosphorylation of the hydrophobic motif on PKB requires catalytically competent PKB (Toker and Newton, 2000). To evaluate the possibility of PKB autophosphorylation on Ser473, we performed the *in-vitro* DNA-PK kinase assay with mono-phosphorylated PKB in presence of 1 μM staurosporine (in which PKB activity is completely blocked while Ser473 kinase activity is resistant (Hill *et al.*, 2001)). PKB autophosphorylation on Ser473 is not occurred even though PKB get phosphorylate on The-308, which imply the active PKB (data not shown). In presence of 1 μM staurosporine where PKB activity is completely inhibited (Hill *et al.*, 2001), DNA-PK still phosphorylates PKB on Ser473 (data not shown). These results perfectly match with the previous report (Hill *et al.*, 2001), indicating that DNA-PK is the Ser473 kinase for PKB.

In the current study, we further provided the evidence that DNA-PK is a responsible Ser473 kinase for PKB by using DNA-PK knockout MEF cells. We show that Ser473 phosphorylation of PKB is impaired in DNA-PK knockout MEF cells following insulin stimulation. Furthermore, similar observation was also obtained from DNA-PK deficient cells while there is no significant effect on Ser473 phosphorylation of PKB in ATM knockout MEF cells, which is another PI3K-like kinase. The treatment of cell with Doxorubicin, DNA-damage reagent, also lead to an increase in Ser473 phosphorylation in DNA-PK wild type MEF cells, which is blocked in DNA-PK knockout MEF cells. Taken together, these data clearly demonstrate that DNA-PK has a novel role in PKB signaling in insulin and DNA-repair signaling pathway. Furthermore, this report supports the role of DNA-PK in Ser473 phosphorylation for regulating PI3K/PKB signaling pathways. And it would provide better information for the clinical utilization of DNA-PK inhibitors.

## ACKNOWLEDGMENTS

We would like to thank Dr. P.A. Jeggo (University of Sussex, UK) for DNA-PK knockout MEFs and Dr. F. W. Alt (Harvard University, USA) for ATM knockout MEFs. This work was supported by the ERC Program (R11-2002-100-02006-0) and the Basic Research Program (R01-2005-000-10240-0, F01-2005-000-10011-0) of the Korea Science & Engineering Foundation grant funded by the Korea government (MOST), by a grant from the National R&D Program for Cancer Control Ministry of Health & Welfare, Republic of Korea. (NO-0720560) and by Research fund of Chungnam National University Hospital in 2006.

## References

[CR1] Alessi DR, Andjelkovic M, Caudwell B, Cron P, Morrice N, Cohen P, Hemmings BA (1996). Mechanism of activation of protein kinase B by insulin and IGF-1. Embo. J..

[CR2] Alessi DR, James SR, Downes CP, Holmes AB, Gaffney PR, Reese CB, Cohen P (1997). Characterization of a 3-phosphoinositide-dependent protein kinase which phosphorylates and activates protein kinase Balpha. Cuir. Biol..

[CR3] Allalunis-Turner MJ, Barron GM, Day RS, Dobler KD, Mirzayans R (1993). Isolation of two cell lines from a human malignant glioma specimen differing in sensitivity to radiation and chemotherapeutic drugs. Radiat. Res..

[CR4] Andjelkovic M, Alessi DR, Meier R, Fernandez A, Lamb NJ, Frech M, Cron P, Cohen P, Lucocq JM, Hemmings BA (1997). Role of translocation in the activation and function of protein kinase B. J. Biol. Chem..

[CR5] Andjelkovic M, Maira SM, Cron P, Parker PJ, Hemmings BA (1999). Domain swapping used to investigate the mechanism of protein kinase B regulation by 3-phosphoinositide-dependent protein kinase 1 and Ser473 kinase. Mol. Cell. Biol..

[CR6] Beamish HJ, Jessberger R, Riballo E, Priestley A, Blunt T, Kysela B, Jeggo PA (2000). The C-terminal conserved domain of DNA-PKcs, missing in the SCID mouse, is required for kinase activity. Nucleic. Acids. Res..

[CR7] Bradford MM (1976). A rapid and sensitive method for the quantitation of microgram quantities of protein utilizing the principle of protein-dye binding. Anal. Biochem..

[CR8] Brazil DP, Yang Z-Z, Hemmings BA (2004). Advances in protein kinase B signalling: AKTion on multiple fronts. Trends in Biochemical Sciences.

[CR9] Burma S, Chen DJ (2004). Role of DNA-PK in the cellular response to DNA double-strand breaks. DNA Repair (Amst).

[CR10] Byrum J, Jordan S, Safrany ST, Rodgers W (2004). Visualization of inositol phosphate-dependent mobility of Ku: depletion of the DNA-PK cofactor InsP6 inhibits Ku mobility. Nucleic. Acids. Res..

[CR11] Caporali S, Levati L, Starace G, Ragone G, Bonmassar E, Alvino E, D’Atri S (2008). AKT is activated in an ATR-dependent manner in response to temozolomide and confers protection against drug-induced cell growth inhibition. Mot Pharmacol..

[CR12] Chan DW, Son SC, Block W, Ye R, Khanna KK, Wold MS, Douglas P, Goodarzi AA, Pelley J T Y, Lavin MF, Lees-Miller SP (2000). Purification and characterization of ATM from human placenta. A manganese-dependent, wortmannin-sensitive serine/threonine protein kinase. J. Biol. Chem..

[CR13] Citterio E, Vermeulen W, Hoeijmakers JH (2000). Transcriptional healing. Cell..

[CR14] Dummler B, Hemmings BA (2007). Physiological roles of PKB/Akt isoforms in development and disease. Biochem. Soc. Trans.

[CR15] Feng J, Park J, Cron R, Hess D, Hemmings BA (2004). Identification of a PKB/Akt hydrophobic motif ser-473 kinase as DNA-dependent protein kinase. J. Biol. Chem..

[CR16] Gottschalk AR, Doan A, Nakamura JL, Stokoe D, Haas-Kogan DA (2005). Inhibition of phosphatidylinositol-3-kinase causes increased sensitivity to radiation through a PKB-dependent mechanism. Int. J. Radiat. Oncol. Biol. Phys..

[CR17] Grana TM, Rusyn EV, Zhou H, Sartor CI, Cox AD (2002). Ras mediates radioresistance through both phosphatidylinositol 3-kinase-dependent and Raf-dependent but mitogen-activated protein kinase/extracellular signal-regulated kinase kinase-independent signaling pathways. Cancer. Res..

[CR18] Gupta AK, Bakanauskas VJ, Cerniglia GJ, Cheng Y, Bernhard EJ, Muschel RJ, McKenna WG (2001). The Ras radiation resistance pathway. Cancer. Res..

[CR19] Gupta AK, Cerniglia GJ, Mick R, Ahmed MS, Bakanauskas VJ, Muschel RJ, McKenna WG (2003). Radiation sensitization of human cancer cells *in vivo* by inhibiting the activity of PI3K using LY294002. Int. J. Radial Oncol. Biol. Phys..

[CR20] Hanakahi LA, Bartlet-Jones M, Chappell C, Pappin D, West SC (2000). Binding of inositol phosphate to DNA-PK and stimulation of double-strand break repair. Cell..

[CR21] Hanakahi LA, West SC (2002). Specific interaction of IP6 with human Ku70/80, the DNA-binding subunit of DNA-PK. Embo. J..

[CR22] Hartley KO, Gell D, Smith GC, Zhang H, Divecha N, Connelly MA, Admon A, Lees-Miller SP, Anderson CW, Jackson SP (1995). DNA-dependent protein kinase catalytic subunit: a relative of phosphatidylinositol 3-kinase and the ataxia telangiectasia gene product. Cell..

[CR23] Hill MM, Andjelkovic M, Brazil DR, Ferrari S, Fabbro D, Hemmings BA (2001). Insulin-stimulated protein kinase B phosphorylation on Ser-473 is independent of its activity and occurs through a staurosporine-insensitive kinase. J. Biol. Chem..

[CR24] Hoeijmakers JH (2001). DNA repair mechanisms. Maturi-tas.

[CR25] Hoeijmakers JH (2001). Genome maintenance mechanisms for preventing cancer. Nature.

[CR26] Hresko RC, Mueckler M (2005). mTOR.RICTOR is the Ser473 kinase for Akt/protein kinase B in 3T3-L1 adipocytes. J. Biol. Chem..

[CR27] Li B, Yuan M, Kim IA, Chang CM, Bernhard EJ, Shu HK (2004). Mutant epidermal growth factor receptor displays increased signaling through the phosphatidylinositol-3 kinase/AKT pathway and promotes radioresistance in cells of astrocytic origin. Oncogene.

[CR28] Li X, Lu Y, Liang K, Liu B, Fan Z (2005). Differential responses to doxorubicin-induced phosphorylation and activation of Akt in human breast cancer cells. Breast. Cancer. Res..

[CR29] Loeb LA (1991). Mutator phenotype may be required for multistage carcinogenesis. Cancer. Res..

[CR30] Ma Y, Lieber MR (2002). Binding of inositol hexakisphosphate (IP6) to Ku but not to DNA-PKcs. J. Biol. Chem..

[CR31] Meek K, Gupta S, Ramsden DA, Lees-Miller SP (2004). The DNA-dependent protein kinase: the director at the end. Immunol. Rev.

[CR32] O’Neill T, Dwyer AJ, Ziv Y, Chan DW, Lees-Miller SP, Abraham RH, Lai JH, Hill D, Shiloh Y, Cantley LC, Rathbun GA (2000). Utilization of oriented peptide libraries to identify substrate motifs selected by ATM. J. Biol. Chem..

[CR33] Park J, Hill MM, Hess D, Brazil DR, Hofsteenge J, Hemmings BA (2001). Identification of tyrosine phosphorylation sites on 3-phosphoinositide-dependent protein kinase-1 and their role in regulating kinase activity. J. Biol. Chem..

[CR34] Quevedo C, Kaplan DR, Derry WB (2007). AKT-1 regulates DNA-damage-induced germline apoptosis in C. elegans. Curr. Biol..

[CR35] Sarbassov DD, Guertin DA, Ali SM, Sabatini DM (2005). Phosphorylation and regulation of Akt/PKB by the rictor-mTOR complex. Science.

[CR36] Stephens L, Anderson K, Stokoe D, Erdjument-Bromage H, Painter GF, Holmes AB, Gaffney PR, Reese CB, McCormick F, Tempst P, Coadwell J, Hawkins PT (1998). Protein kinase B kinases that mediate phosphatidylinositol 3,4,5-trisphosphate-dependent activation of protein kinase B. Science.

[CR37] Stokoe D, Stephens LR, Copeland T, Gaffney PR, Reese CB, Painter GF, Holmes AB, McCormick F, Hawkins PT (1997). Dual role of phosphatidylinositol-3,4,5-trisphosphate in the activation of protein kinase B. Science.

[CR38] Taccioli GE, Amatucci AG, Beamish HJ, Gell D, Xiang XH, Torres Arzayus MI, Priestley A, Jackson SP, Marshak Rothstein A, Jeggo PA, Herrera VL (1998). Targeted disruption of the catalytic subunit of the DNA-PK gene in mice confers severe combined immunodeficiency and radiosensitivity. Immunity.

[CR39] Toker A, Newton AC (2000). Akt/protein kinase B is regulated by autophosphorylation at the hypothetical PDK-2 site. J. Biol. Chem..

[CR40] van Gent DC, Hoeijmakers JH, Kanaar R (2001). Chromosomal stability and the DNA double-stranded break connection. Nat. Rev. Genet.

[CR41] Viniegra JG, Martinez N, Modirassari P, Losa JH, Parada Cobo C, Lobo VJ, Luquero CI, Alvarez-Vallina L, Ramon y Cajal S, Rojas JM, Sanchez-Prieto R (2005). Full activation of PKB/Akt in response to insulin or ionizing radiation is mediated through ATM. J. Biol. Chem..

[CR42] Weterings E, Chen DJ (2007). DNA-dependent protein kinase in nonhomologous end joining: a lock with multiple keys?. J. Cell. Biol..

[CR43] Weterings E, van Gent DC (2004). The mechanism of non-homologous end-joining: a synopsis of synapsis. DNA Repair (Amst).

[CR44] Yang ZZ, Tschopp O, Baudry A, Dummler B, Hynx D, Hemmings BA (2004). Physiological functions of protein kinase B/Akt. Biochem. Soc. Trans.

